# ChatGPT provides inconsistent risk-stratification of patients with atraumatic chest pain

**DOI:** 10.1371/journal.pone.0301854

**Published:** 2024-04-16

**Authors:** Thomas F. Heston, Lawrence M. Lewis

**Affiliations:** 1 Department of Family Medicine, University of Washington School of Medicine, Seattle, Washington, United States of America; 2 Department of Medical Education and Clinical Sciences, Washington State University, Spokane, Washington, United States of America; 3 Department of Emergency Medicine, Washington University, Saint Louis, Missouri, United States of America; Wiltse Memorial Hospital, REPUBLIC OF KOREA

## Abstract

**Background:**

ChatGPT-4 is a large language model with promising healthcare applications. However, its ability to analyze complex clinical data and provide consistent results is poorly known. Compared to validated tools, this study evaluated ChatGPT-4’s risk stratification of simulated patients with acute nontraumatic chest pain.

**Methods:**

Three datasets of simulated case studies were created: one based on the TIMI score variables, another on HEART score variables, and a third comprising 44 randomized variables related to non-traumatic chest pain presentations. ChatGPT-4 independently scored each dataset five times. Its risk scores were compared to calculated TIMI and HEART scores. A model trained on 44 clinical variables was evaluated for consistency.

**Results:**

ChatGPT-4 showed a high correlation with TIMI and HEART scores (r = 0.898 and 0.928, respectively), but the distribution of individual risk assessments was broad. ChatGPT-4 gave a different risk 45–48% of the time for a fixed TIMI or HEART score. On the 44-variable model, a majority of the five ChatGPT-4 models agreed on a diagnosis category only 56% of the time, and risk scores were poorly correlated (r = 0.605).

**Conclusion:**

While ChatGPT-4 correlates closely with established risk stratification tools regarding mean scores, its inconsistency when presented with identical patient data on separate occasions raises concerns about its reliability. The findings suggest that while large language models like ChatGPT-4 hold promise for healthcare applications, further refinement and customization are necessary, particularly in the clinical risk assessment of atraumatic chest pain patients.

## Introduction

The feasibility of employing artificial intelligence (AI) to enhance healthcare has become possible due to revolutionary advancements in neural network architecture. The initial application of neural networks to machine learning occurred in the 1940s with the development of a single-neuron model [1]. Over the decades, computer neural networks gradually increased in complexity. However, it is only in recent years that advances in computer processing speeds, combined with the expansion of the Internet, have led to programs capable of directly communicating with humans in natural language [2]. A significant milestone was the development of the transformer architecture in 2017, which enhanced the language understanding of neural networks by enabling the interpretation of word context [3]. Subsequently, in 2018, the introduction of the generative pre-trained transformer (GPT) model marked a breakthrough in generating coherent text [4].

Since the introduction of GPT-1 in 2018, this model has undergone continuous improvements. As a result, an Internet-based chatbot, ChatGPT-4, now facilitates natural conversations with humans in various languages, encompassing spoken, archaic, and software coding languages. All versions of these GPT large language models (LLMs) incorporate attention mechanisms, enabling the model to concentrate on specific inputs for learning. This feature allows the neural network to learn from raw, unlabeled data, a significant advancement from earlier models that relied on labeled data, necessitating pre-processing and interpretation. While ChatGPT-4 demonstrates proficiency in conducting general conversations in multiple languages, its capacity for medical reasoning and understanding remains to be thoroughly assessed. Several studies have indicated ChatGPT’s competence in executing single medical task commands, such as answering multiple-choice questions from exams like the United States Medical Licensing Exam [5, 6] and various medical specialty exams [6]. However, ChatGPT-4 struggles with logical questions [7] and occasionally fabricates responses [8]. It also can produce convincing yet misleading text with notable inaccuracies [9].

Diagnostic imaging in medicine has been a prominent area for AI application. Recent studies evaluating AI’s ability to identify specific or a limited number of pathological conditions demonstrate that AI’s accuracy rivals that of trained physicians in interpreting certain radiographic procedures [10]. However, it does not match the precision achieved through double-reading practices [11]. Clinical decision-making and risk stratification involve more complex considerations, requiring inputs from patient history, examination, laboratory results, and imaging studies. Despite this complexity, numerous studies indicate that AI, particularly machine learning, matches or surpasses existing standard risk-stratification tools for conditions such as transcatheter aortic valve implantation [12], surgical risk assessment [13], and cardiovascular risk prediction [14–17] Most of these studies utilized machine learning to amalgamate and analyze labeled data from clinical registries, including outcome data, into their predictive models.

Chest pain is a frequent chief complaint in emergency departments (ED), often associated with severe medical conditions but predominantly of benign origin. Physicians’ caution has led to the hospitalization of many patients to exclude acute coronary syndrome (ACS), even in cases without significant heart disease. This overutilization of resources has spurred the development of precise risk-stratification protocols to accurately identify low-risk patients who do not require admission and rapidly assess and intervene in high-risk patients. The TIMI score is one such protocol, a seven-item tool derived and validated on patients with unstable angina (UA) or non-ST-segment myocardial infarction (NSTEMI) in two separate clinical trials comparing clinical outcomes in subjects receiving unfractionated heparin to those receiving enoxaparin. The predicted outcome of interest was all-cause mortality, new or recurrent myocardial infarction (MI), or severe recurrent ischemia (necessitating urgent revascularization) within 14 days post-randomization [18].

Although the TIMI score was initially designed for patients with a clinical diagnosis of UA or NSTEMI receiving anticoagulation, it has subsequently been shown to predict 30-day to 6-week major adverse cardiac events (MACE) in ED patients with atraumatic chest pain of suspected cardiac origin [19, 20]. MACE was defined as acute MI, coronary revascularization, or death from any cause.

Another protocol for acute non-traumatic chest pain is the HEART score [21], a five-item tool initially derived from a small patient cohort in the ED to predict MACE over three months. Subsequent studies have confirmed the HEART score as a robust predictor of 1-month to six-week MACE risk [19, 20, 22, 23], with scores less than or equal to 3 identifying a low-risk group with about a 2% incidence of MACE. Despite their utility, these tools have faced scrutiny regarding their sensitivity, particularly in the case of TIMI [24,25].

By testing ChatGPT-4’s capacities through simulated acute chest pain cases, this study aims to uncover strengths and vulnerabilities to guide responsible AI development. Specifically, we evaluate ChatGPT-4’s capability in risk-stratifying simulated patients with acute nontraumatic chest pain, comparing its performance with established tools like the TIMI and HEART scores. Additionally, ChatGPT-4 was tested on more complex simulated cases to determine key variables it deems crucial for risk stratification and to verify the consistency of its responses when presented with identical data on multiple occasions.

## Materials and methods

### Data generation

Case studies were randomly generated using a Python software program. All cases were computer-simulated; no actual patient data was involved. Three datasets of simulated case studies were created.

The first dataset included the seven TIMI score variables for UA or NSTEMI [18]. For effective interaction with ChatGPT-4, the variables were encoded in a binary manner as follows: age ≥ 65 years (yes/no); ≥ 3 coronary artery disease (CAD) risk factors (yes/no); known CAD (yes/no); aspirin use within the past seven days (yes/no); at least two episodes of severe angina in the past 24 hours (yes/no); ECG ST changes ≥ 0.5 mm (yes/no); and positive cardiac marker (yes/no).

The second dataset comprised the five HEART score variables for major cardiac events [21], encoded as follows: history (slightly suspicious, moderately suspicious, or highly suspicious); ECG (normal, non-specific repolarization disturbance, or significant ST deviation); age categories (<45 years, 45–64 years, or >64 years); risk factors (no known risk factors, one or two risk factors, or three or more risk factors); and initial troponin (normal, one to three times the normal limit, or more than three times the normal limit).

The third dataset of simulated cases included forty-four randomized variables pertinent to the acute presentation of non-traumatic chest pain. These variables were selected to represent typical clinical findings in acute nontraumatic chest pain patients and for their value in helping to risk-stratify patients with chest pain as to the likelihood of a cardiovascular underlying cause. This dataset focused solely on history and physical examination findings, excluding any test results. The variables encompassed age (ranging from 40 to 90 years), duration of pain (in minutes), pain severity level (scaled from 1 to 10), gender (Male/Female), race (African American or non-African American), and a series of binary variables coded as 0 (no) or 1 (yes). These binary variables included: substernal chest pain, heavy pain, burning pain, pain triggered by exertion or stress, pain alleviation by rest or nitroglycerin, exacerbation of pain when lying down, pain intensification with deep breathing, current aspirin use, current blood pressure medication use, current nonsteroidal anti-inflammatory medication use, current statin medication use, current insulin use, cocaine use, moderate to heavy alcohol use, smoking status, history of hypertension, history of myocardial infarction, diagnosed coronary artery disease, diabetes history, stroke history, symptoms of nausea, dyspnea, palpitations, dizziness, marital status, family history of coronary artery disease, hypotension on examination, hypertension on examination, bradycardia on examination, tachycardia on examination, fever on examination, tachypnea on examination, hypoxia on examination, weak pulse, irregular heart rhythm, abnormal lung sounds upon auscultation, pain reproducible upon palpation, cardiac murmur detected during auscultation, and presence of edema.

### ChatGPT-4 analysis

The three datasets underwent individual processing. Initially, each dataset was uploaded to ChatGPT-4 (version dated September 25, 2023) for Advanced Data Analysis. A standardized set of prompts was employed to facilitate interaction with ChatGPT-4. The initial prompt directed ChatGPT-4 to assign a risk score for acute coronary syndrome to each case. The scoring scale for the first dataset corresponded to the TIMI scale, ranging from 0 to 7. For the second dataset, the scale was aligned with the HEART scale, ranging from 0 to 10. The final dataset utilized a scale from 0 to 100. Subsequently, ChatGPT-4 was instructed to disclose the weighting assigned to each variable in the calculation of its risk scores. Specifically, for the third dataset, ChatGPT-4 was required to assign a weight to each of the 44 variables, with the option of assigning a weight of zero.

Additionally, for the third set of simulated cases, ChatGPT-4 received an open-ended prompt to specify the first diagnostic test it would recommend in the emergency department for each case without any specific guidelines or limitations.

Each dataset was presented to ChatGPT-4 five separate times, prompting the AI to assign a risk score to each case and then give the weight assigned to each variable. The datasets remained unchanged, ensuring the same data was presented during each of the five sessions. ChatGPT-4 generated five distinct models for each of the three datasets, resulting in a total of 15 models. A new chat session was initiated for each model creation. For the third dataset, each of the five AI models also included a recommendation for the initial diagnostic test to order in the emergency department for each case. The models were labeled sequentially as 1.1, 1.2, 1.3, 1.4, and 1.5 for the first dataset, with a similar naming convention applied to the models of the second and third datasets. The TIMI score for each simulated patient in the first dataset and the HEART score for each in the second dataset were calculated only after the ChatGPT-4 models had completed their risk assessments.

Consequently, each case of the first dataset included the seven TIMI variables, the TIMI score, and five AI-generated risk scores (one from each of the five ChatGPT-4 models). Each case of the second dataset included the five HEART variables, the HEART score, and five AI-generated risk scores. Each case of the third dataset consisted of forty-four variables, five AI-generated risk scores, and five “first test” recommendations (one from each ChatGPT-4 model). The weights assigned to each variable by each of the 15 ChatGPT-4 generated models were recorded.

### Statistical analysis

The three final datasets, which included the outputs from ChatGPT-4, underwent evaluation using IBM SPSS Statistics (Version 29). Microsoft Excel was employed for the computation of average weighting scores. Pearson’s correlation coefficient and R-squared values were calculated to compare the risk scores generated by each model. The recommended initial diagnostic tests were categorized by gender and race, followed by a test of proportions to examine potential differences. The Kruskal-Wallis test was utilized to assess whether the models created by ChatGPT-4 for each dataset exhibited statistical differences. A single sample t-test was used to determine if the weights assigned by the five ChatGPT-4 models for the history and physical only dataset for sex were different than an expected weight of zero. The study’s design and analysis adhered to the SAMPL guidelines [26].

## Results

ChatGPT-4 initially encountered challenges in assigning risk scores consistent with the TIMI and HEART scales, often producing scores on condensed or expanded scales. Consequently, the prompting approach was modified, directing ChatGPT-4 to provide a risk score within a range of 0 to 100. In this format, ChatGPT-4 effectively assigned risk scores without difficulty. These scores were subsequently recalibrated to align with the TIMI and HEART scales.

In the case of the third dataset, which encompassed forty-four variables based on a simulated patient’s history and physical examination, ChatGPT-4 initially tended to use shortcuts in weight assignment. It often stated that it was setting a default weight for several variables for reasons of "convenience" or "brevity." To address this, the prompt was adjusted to compel ChatGPT-4 to independently assess each variable and assign an appropriate weight in formulating the risk score and its recommendation for the first diagnostic test in the emergency department. The allowance of negative weights or weights of zero was explicitly stated.

### TIMI dataset

**Fig 1:**
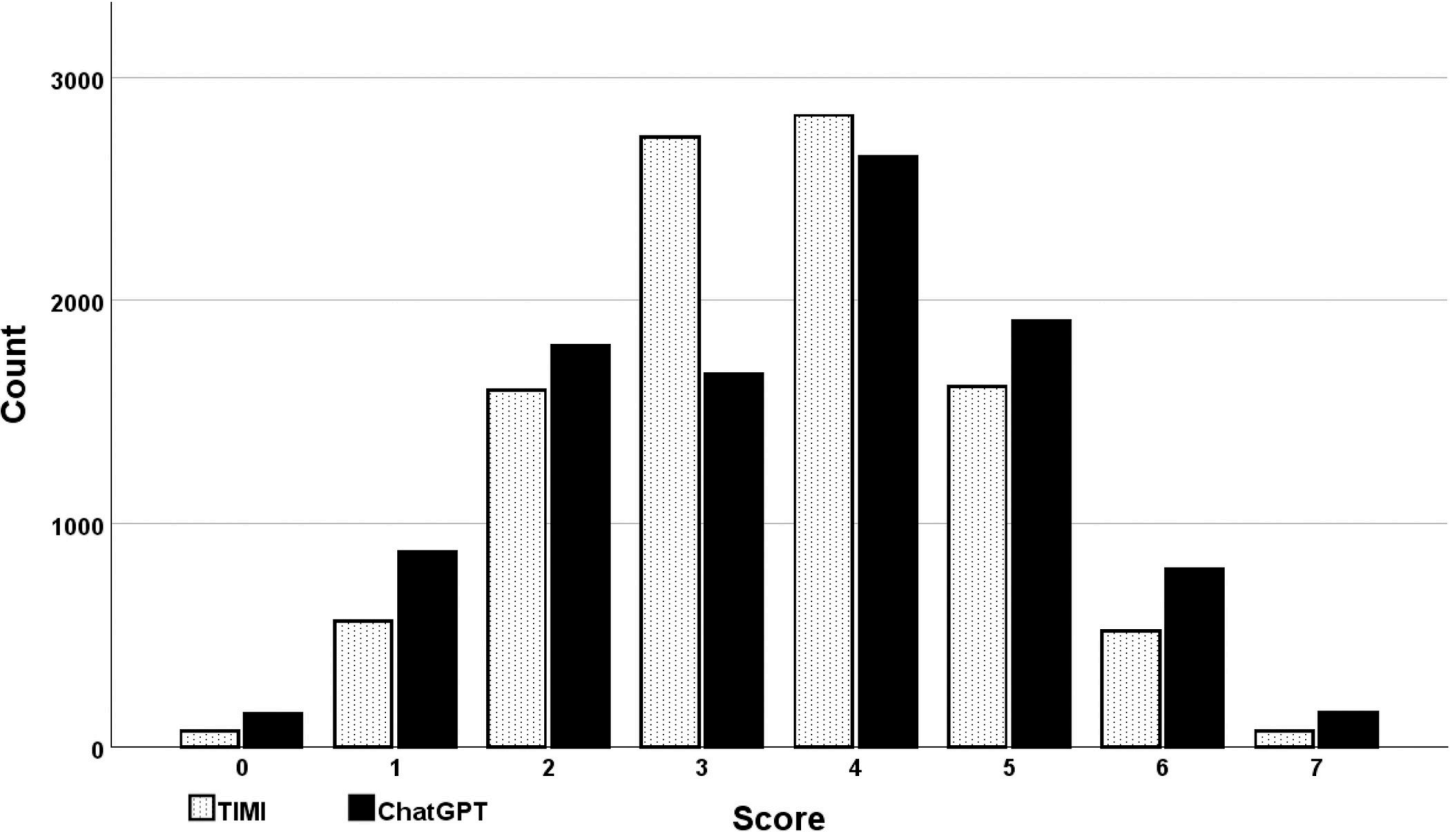
Histogram of TIMI and ChatGPT-4 scores. Both the TIMI and ChatGPT-4 scores demonstrated a normal distribution. However, the distribution of ChatGPT-4 scores was broader than that of the TIMI scores.

A total of 10,000 simulated cases were generated for the TIMI dataset. The distribution of TIMI scores was normal (Fig 1). The correlation coefficients between the TIMI scores and the five ChatGPT-4 models were notably high and, in all instances, statistically significant, each exhibiting a p-value of < 0.001 (Table 1). Overall, the correlation between the TIMI scores and ChatGPT-4 was 0.898 (p < 0.001).

**Table 1:** TIMI dataset correlation matrix.

	TIMI	Model 1.1	Model 1.2	Model 1.3	Model 1.4
**Model 1.1**	0.930	-	-	-	-
**Model 1.2**	0.900	0.921	-	-	-
**Model 1.3**	0.899	0.916	0.949	-	-
**Model 1.4**	0.851	0.931	0.862	0.877	-
**Model 1.5**	0.908	0.894	0.936	0.958	0.815

Pearson correlation coefficients were 0.815 or greater in all cases. All correlation coefficients were statistically significant with p < 0.001.

**Fig 2:**
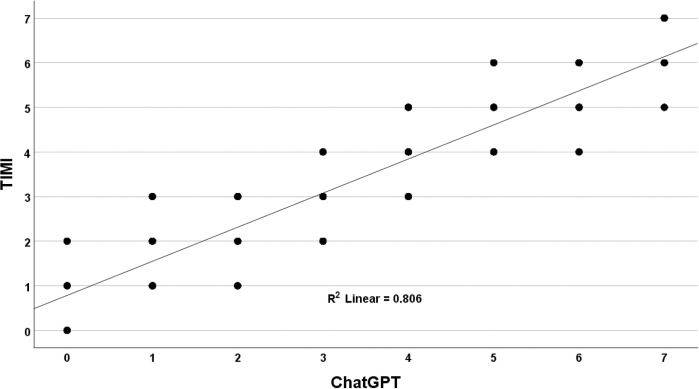
Comparison of TIMI with ChatGPT-4. The correlation between TIMI risk score and ChatGPT-4 risk estimates over 5 simulated runs. While the overall correlation was high (R-squared = 0.806), ChatGPT-4’s scores demonstrated broad variability across the distribution relative to the TIMI benchmark standard.

While the correlations were consistently high, the analysis revealed a broad distribution in the comparison of ChatGPT-4 scores with TIMI scores. For TIMI scores at the extremes of zero and seven, as expected, there was perfect alignment with the ChatGPT-4 models. This was anticipated since these models evaluated only seven variables; for a TIMI score of zero, all variables would be negative, leading to a cumulative weighting of zero by ChatGPT-4. Conversely, a TIMI score of seven, where all variables would be positive, would result in the maximum cumulative weighting by ChatGPT-4. The mean (standard error) for TIMI scores was 3.50 (0.013), and for ChatGPT-4, it was 3.55 (0.015), a statistically significant difference as determined by a paired t-test (p < 0.001). Across the 5 independent simulation runs, ChatGPT-4 assigned a different score than TIMI in 45% of the cases, demonstrating the random divergence clinicians would encounter if using ChatGPT-4 for risk stratification. ChatGPT-4 produced three to four different scores for each fixed TIMI score ranging from one to six (Fig 2).

ChatGPT-4’s performance relative to the TIMI benchmark showed less variability at low and high scores. For a TIMI score of 1, ChatGPT-4’s average (avg) and standard deviation (sd) was 0.98 (0.45). For middle scores from 2 to 5, ChatGPT-4’s avg (sd) were 1.85 (0.64), 3.04 (0.80), 4.15 (0.68), and 5.12 (0.61). For a TIMI score of 6, ChatGPT-4’s avg (sd) was 6.07 (0.46). The greater variability for mid-range TIMI scores suggests that ChatGPT-4 performs worse in predicting outcomes for intermediate-risk patients than low- and high-risk patients based on TIMI benchmark standards.

The weights assigned to each variable by the five ChatGPT-4 models were found to be statistically similar, as indicated by a p-value of 0.406 according to the Kruskal-Wallis test. However, despite this statistical similarity, the models differed in the specific weights assigned to each variable. The detailed weights allocated to each TIMI variable by each of the five ChatGPT-4 models are presented in Table 2.

**Table 2:** Weights assigned to the TIMI variables by the five ChatGPT-4 models.

TIMI Variables	TIMI	MODEL 1.1	MODEL 1.2	MODEL 1.3	MODEL 1.4	MODEL 1.5
Age > = 65 years	1.00	0.70	1.40	1.05	0.70	1.05
CAD risk factors > = 3	1.00	1.05	1.05	1.40	1.05	1.40
Known CAD	1.00	1.40	1.75	1.75	1.40	1.75
ASA use past 7 days	1.00	0.35	0.35	0.35	-0.35	0.70
Severe angina (> = 2 episodes in 24 hrs)	1.00	1.05	0.70	1.05	1.40	0.70
ECG ST changes > = 0.5 mm	1.00	1.40	1.05	0.70	1.40	0.70
Positive cardiac marker	1.00	1.05	0.70	0.70	1.40	0.70
**Total**	7.00	7.00	7.00	7.00	7.00	7.00

A positive cardiac marker refers to an elevated CKMB or troponin level. CAD, coronary artery disease; ASA, aspirin; ECG, electrocardiogram.

### HEART dataset

**Fig 3:**
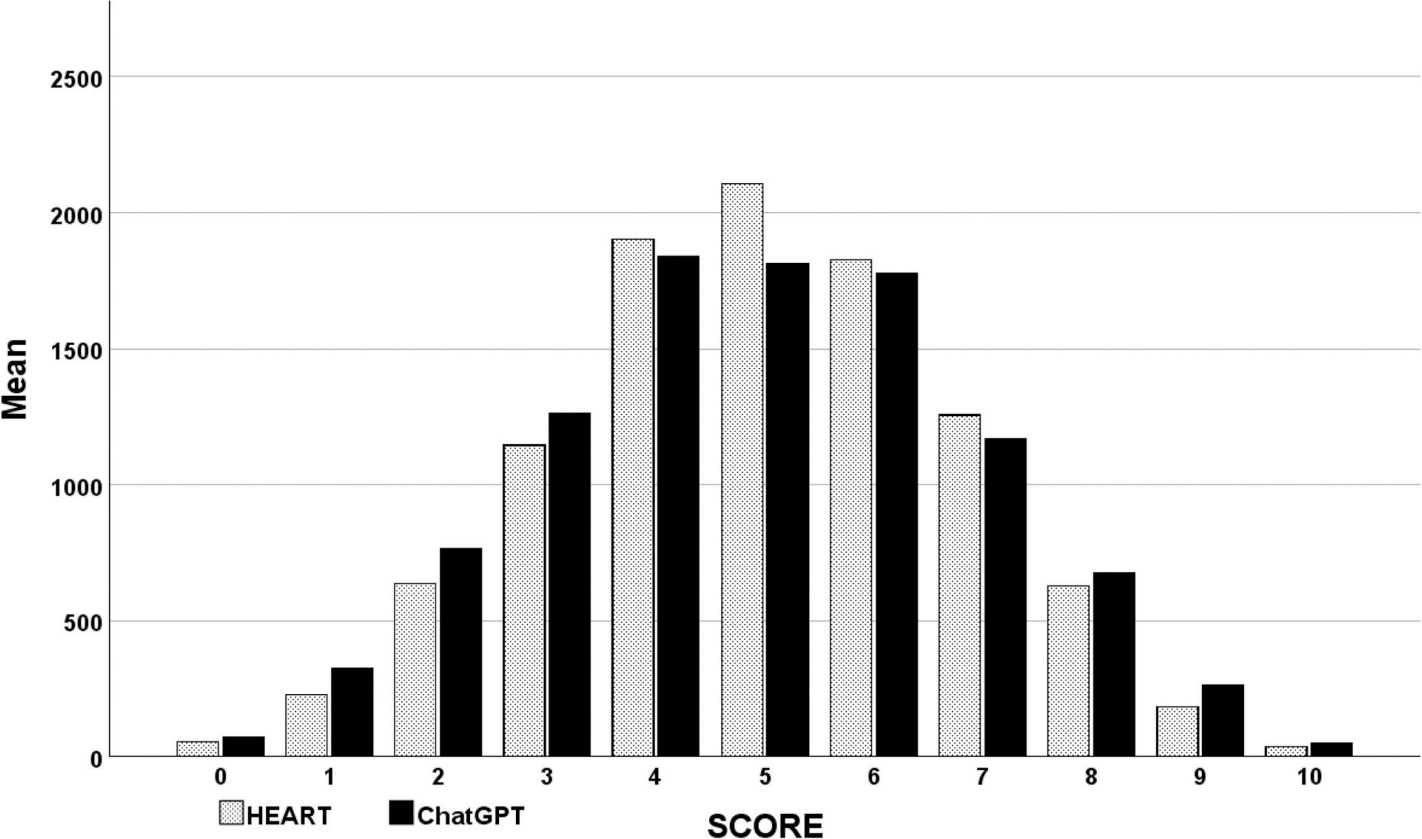
Histogram of HEART and ChatGPT-4 scores. Both the HEART and ChatGPT-4 scores exhibited a normal distribution. However, the distribution of ChatGPT-4 scores was broader than that of the HEART scores.

A total of 10,000 simulated cases were generated for the HEART dataset. The distribution of HEART scores followed a normal pattern (Fig 3). The correlation coefficients between the HEART scores and the five ChatGPT-4 models were consistently high and, in every instance, statistically significant, each exhibiting a p-value of less than 0.001 (Table 3). The overall correlation between the HEART scores and ChatGPT-4 was 0.928 (p < 0.001).

**Table 3:** HEART dataset correlation matrix.

	HEART	Model 2.1	Model 2.2	Model 2.3	Model 2.4
Model 2.1	0.912	-	-	-	-
Model 2.2	0.936	0.961	-	-	-
Model 2.3	0.956	0.874	0.865	-	-
Model 2.4	0.946	0.950	0.951	0.879	-
Model 2.5	0.953	0.979	0.981	0.912	0.947

Pearson correlation coefficients were 0.865 and greater in all cases. All correlation coefficients were statistically significant with p < 0.001.

**Fig 4:**
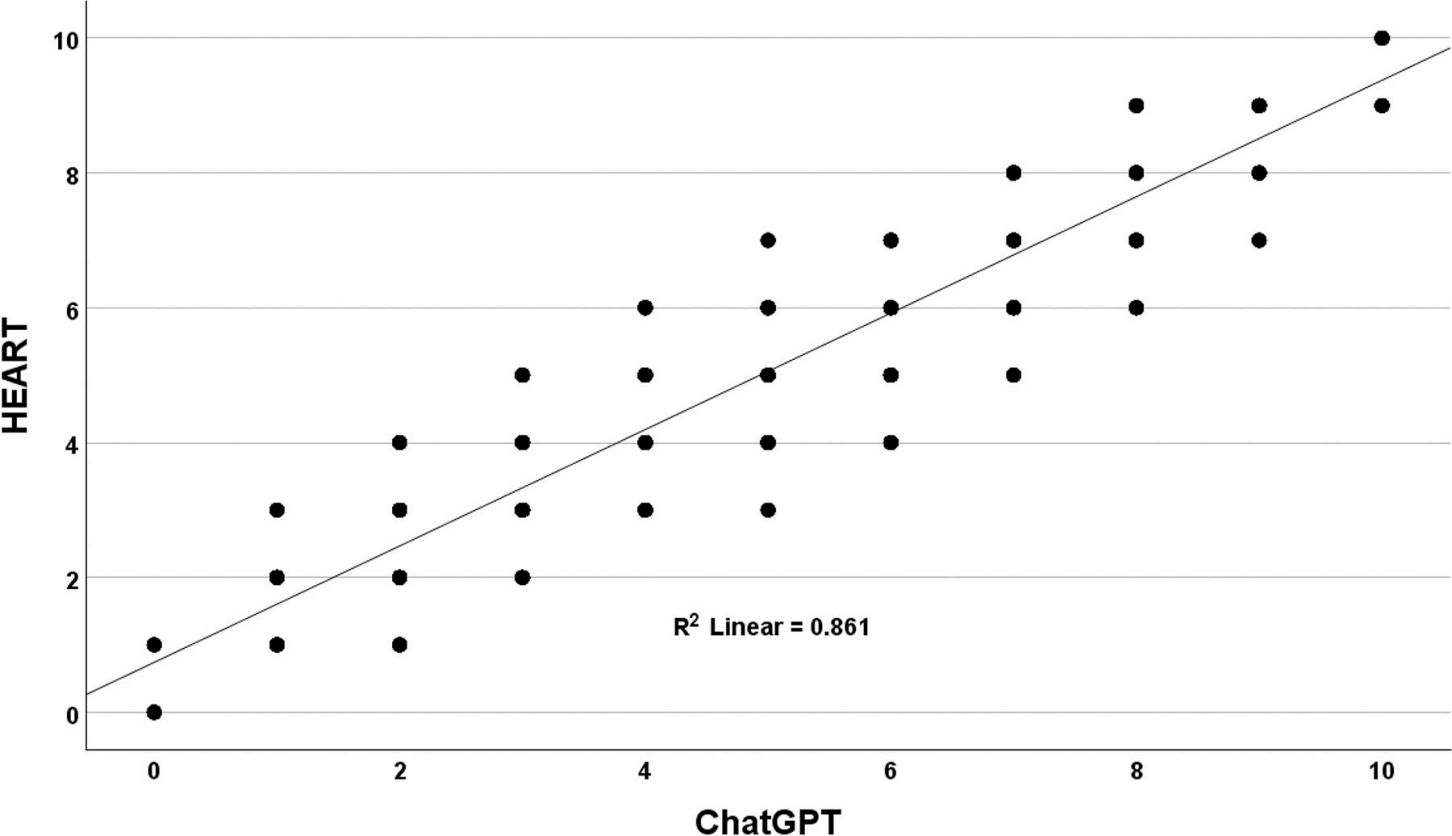
Comparison of HEART with ChatGPT-4. The correlation between HEART risk score and ChatGPT-4 scores over 5 simulated runs. While the overall correlation was high (R-squared = 0.861), ChatGPT-4 demonstrated broad score variability across the distribution relative to the HEART benchmark standard.

Mirroring the TIMI analysis, the correlations between the ChatGPT-4 models and the HEART scores were consistently high. However, the ChatGPT-4 data exhibited a much broader distribution compared to the HEART scores. Perfect alignment with the ChatGPT-4 models was observed for HEART scores at the extremes of zero and ten, which was anticipated as these models evaluated ten variables. The mean (standard deviation [sd]) for the HEART score was 4.99 (0.018), while for ChatGPT-4, it was 4.92 (0.020), a difference statistically significant by a paired t-test (p < 0.001). Across the 5 independent simulation runs, ChatGPT-4 assigned a different risk score than HEART in 48% of cases. This demonstrates the degree of random divergence clinicians could encounter using ChatGPT-4 for cardiac risk assessments. ChatGPT-4 produced a wide variation in outputs for HEART scores from one to nine (Fig 4).

Similar to the findings for TIMI, ChatGPT-4’s variability relative to the HEART benchmark was less at the low and high-end scores. For a HEART score of 1, ChatGPT-4’s mean (sd) was 1.04 (0.413). For HEART scores from 2 to 8, ChatGPT-4 displayed greater variability with the mean (sd) equal to: 1.91 (0.591), 2.94 (0.719), 3.93 (0.755), 4.92 (0.802), 5.94 (0.776), 6.94 (0.740), 7.88 (0.647). At the high-end HEART score of 9, ChatGPT-4’s mean (sd) was 8.98 (0.444).

HEART variables are categorized into three risk groups: low, moderate, and high. Utilizing a low-risk HEART score as the benchmark, ChatGPT-4 demonstrated a sensitivity of 88%, a specificity of 93%, a positive predictive value of 76%, and a negative predictive value of 97%, as detailed in Table 4. The overall agreement between HEART and ChatGPT-4, encompassing all 10,000 simulated patients, the three risk categories, and the five models, varied between 39% to 89%, as shown in Table 5.

**Table 4:** Performance of ChatGPT-4 in predicting a low-risk HEART score.

	HEART Low-Risk	HEART Moderate to Severe Risk	Total
ChatGPT Low-Risk	1824	581	2405
ChatGPT Moderate to Severe Risk	238	7357	7595
Total	2062	7938	10000

The positive predictive value of a low-risk ChatGPT-4 score indicating a low-risk HEART score was 76%.

**Table 5:** Overall agreement between HEART and ChatGPT-4 at each of the three risk categories.

	Risk	Agreement
Low	0.9% - 1.7%	75%
Moderate	12% - 16.6%	89%
High	50% - 65%	39%

The agreement of HEART and ChatGPT-4 assigned risk categories ranged from 39% to 89%.

The weights assigned to each HEART variable by the five ChatGPT-4 models were statistically similar (p = 0.277 by Kruskal-Wallis). However, the various models set notably different weights for each variable. (Table 6).

**Table 6:** Weights assigned to the HEART variables by the five ChatGPT-4 models.

	HEART	MODEL 2.1	MODEL 2.2	MODEL 2.3	MODEL 2.4	MODEL 2.5
Slightly suspicious history	0.00	0.43	0.00	0.41	0.47	0.00
Moderately suspicious history	1.00	1.29	1.25	1.22	1.41	1.25
Highly suspicious history	2.00	2.14	2.50	2.03	2.34	2.50
Normal ECG	0.00	0.00	0.00	0.00	0.00	0.00
Nonspecific repolarization	1.00	0.86	1.04	0.81	0.94	1.04
Significant ST changes	2.00	1.71	2.08	2.03	1.88	2.08
Age under 45	0.00	0.00	0.00	0.00	0.00	0.00
Age 45 to 64	1.00	0.86	0.63	0.81	0.94	0.83
Age over 64	2.00	1.71	1.25	1.62	1.41	1.67
No known risks	0.00	0.00	0.00	0.00	0.00	0.00
One or two risk factors	1.00	0.86	0.83	0.81	0.94	0.63
Three or more risk factors	2.00	1.71	1.67	1.62	1.88	1.25
Normal troponin	0.00	0.00	0.00	0.00	0.00	0.00
Troponin 1-3x normal	1.00	1.29	1.25	1.22	0.94	1.25
Troponin > 3x normal	2.00	2.14	2.50	2.43	1.88	2.50
Total	15	15	15	15	15	15

Although not statistically different, the five ChatGPT-4 models assigned different weights to each HEART variable.

### History and physical-only dataset

For the history and physical-only dataset, 10,000 simulated cases were generated. While the distribution of these scores displayed a slight skewness with a longer tail towards lower scores, it was predominantly normal.

**Fig 5:**
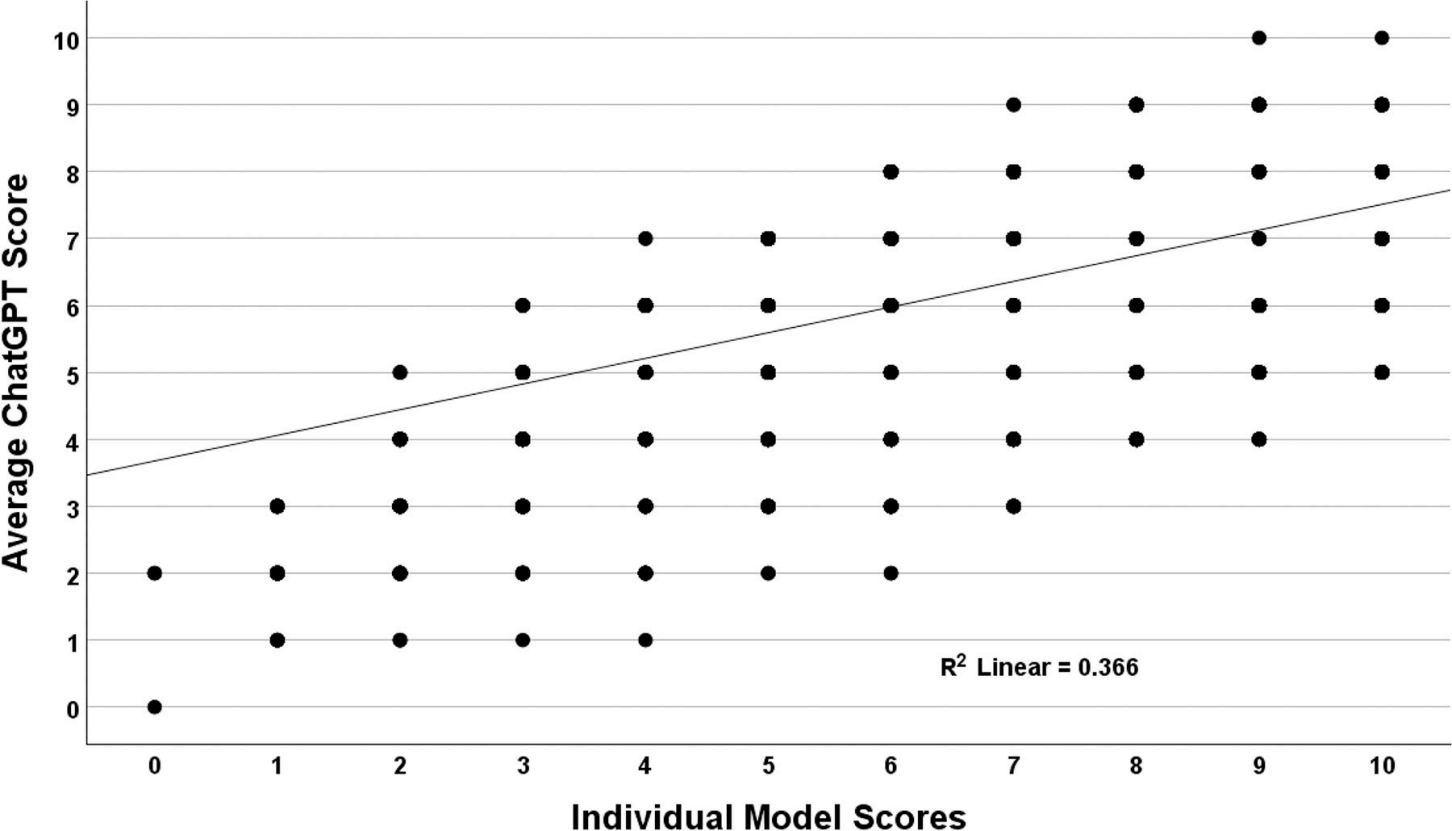
Individual model scores compared to average scores for the history and physical-only dataset. There was a poor correlation between the individual model scores and the average ChatGPT-4 score, consistent with wide variation between the models.

A scatterplot comparing the individual model scores with the average score (used as a surrogate Gold standard) was generated to understand the distribution of risk scores across the five models (Fig 5). While individual correlations with the average risk score were substantial, there was a large variation across the various models (r = 0.605, R-squared = 0.366). To illustrate, for an average score of four, the scores of the individual models ranged from two to nine.

Similar to the HEART and TIMI datasets, variation relative to the benchmark was greatest for the middle-range scores. The model scores (sd) were: 1 (0.919), 2 (1.102), 3 (1.334), 4 (1.627), 5 (1.943), 6 (2.018), 7 (1.687), 8 (1.295), 9 (0.953), and 10 (0.658).

On average, age received the highest weight from the ChatGPT-4 models, contributing 8% to the overall risk score. Similarly, the Diamond and Forrester criteria for chest pain (pain precipitated by exertion or stress, pain relieved by rest or nitroglycerin, and substernal location) were assigned substantial weights [27]. Pain precipitated by exertion or stress contributed 5.5%, pain relieved by rest or nitroglycerin contributed 5.1%, and a substernal location of pain contributed 5%. Pain reproducible on palpation and burning pain were protective factors, decreasing the cardiac risk scores (Table 7).

**Table 7:** Weights assigned by the history and physical dataset.

	Model 1	Model 2	Model 3	Model 4	Model 5	Average
Age	0.093	0.063	0.111	0.060	0.071	0.080
Pain precipitated by exertion or stress	0.037	0.057	0.089	0.048	0.043	0.055
Pain relieved by rest or NTG	0.037	0.057	0.078	0.042	0.043	0.051
Heavy pain	0.037	0.044	0.078	0.042	0.050	0.050
Substernal chest pain	0.047	0.051	0.089	0.006	0.057	0.050
Duration of pain in minutes	0.023	0.032	0.067	0.054	0.035	0.042
Pain level of severity	0.047	0.025	0.067	0.036	0.028	0.041
Male	0.009	0.032	0.056	0.030	0.035	0.032
Weak pulse on exam	0.023	0.019	0.011	0.036	0.043	0.026
Irregular heart rhythm on exam	0.023	0.019	0.011	0.024	0.050	0.025
Uses cocaine	0.014	0.019	0.011	0.048	0.021	0.023
Currently tachycardic on exam	0.023	0.019	0.011	0.024	0.035	0.023
History of diagnosed CAD	0.019	0.019	0.011	0.042	0.021	0.022
Previous myocardial infarction	0.014	0.019	0.011	0.042	0.021	0.022
Currently hypoxic on exam	0.023	0.019	0.011	0.024	0.028	0.021
Currently on insulin	0.023	0.019	0.011	0.030	0.021	0.021
History of hypertension	0.019	0.019	0.011	0.024	0.028	0.020
History of diabetes	0.019	0.019	0.011	0.030	0.021	0.020
Edema present on exam	0.023	0.019	0.011	0.018	0.028	0.020
Currently tachypneic on exam	0.023	0.019	0.011	0.018	0.028	0.020
Currently hypotensive on exam	0.023	0.019	0.011	0.024	0.021	0.020
Currently experiencing dyspnea	0.023	0.019	0.011	0.024	0.021	0.020
Currently on a statin medication	0.023	0.019	0.011	0.024	0.021	0.020
Currently on aspirin	0.023	0.019	0.011	0.024	0.021	0.020
Current smoker	0.014	0.019	0.011	0.030	0.021	0.019
Family history of CAD	0.019	0.019	0.011	0.024	0.021	0.019
Currently hypertensive on exam	0.023	0.019	0.011	0.018	0.021	0.019
Currently bradycardic on exam	0.023	0.019	0.011	0.018	0.021	0.019
Currently experiencing dizziness	0.023	0.019	0.011	0.018	0.021	0.019
Currently experiencing nausea	0.023	0.019	0.011	0.018	0.021	0.019
Currently experiencing palpitations	0.023	0.019	0.011	0.018	0.021	0.019
Currently on a BP medication	0.023	0.019	0.011	0.018	0.021	0.019
History of stroke	0.019	0.019	0.011	0.018	0.021	0.018
Currently on an NSAID	0.023	0.019	0.011	0.012	0.021	0.017
Currently married	0.023	0.019	0.011	0.006	0.021	0.016
Murmur present on cardiac auscultation	0.014	0.019	0.011	0.012	0.021	0.015
Moderate to heavy alcohol use	0.014	0.019	0.011	0.012	0.021	0.015
Pain worse with deep breath	0.014	0.019	0.011	0.012	0.021	0.015
Pain worse with lying down	0.014	0.019	0.011	0.012	0.021	0.015
African American	0.005	0.013	0.022	0.012	0.014	0.013
Currently febrile on exam	0.023	0.019	0.011	0.012	-0.014	0.010
Abnormal lung sounds on auscultation	0.014	0.019	0.011	0.012	-0.021	0.007
Pain reproducible on palpation	0.014	0.019	0.011	-0.030	-0.057	-0.009
Burning type of pain	-0.023	-0.019	-0.033	-0.030	-0.035	-0.028
Total	1.000	1.000	1.000	1.000	1.000	1.000

NTG, nitroglycerin; CAD, coronary artery disease; BP, blood pressure; NSAID, nonsteroidal anti-inflammatory drug.

### Diagnoses and recommendations for initial test

ChatGPT-4 was directed to provide the most probable diagnosis and the optimal initial test to be ordered in the emergency department for the third dataset, which contained the history and physical variables. Notably, the models provided a wide range of diagnoses, often with slight variations in wording. For instance, one model might suggest "acute coronary syndrome," while another might use the abbreviation "ACS." To streamline data analysis, these diagnoses were categorized into specific diagnostic groups: cardiovascular, pulmonary, gastrointestinal, musculoskeletal, or unknown.

**Fig 6:**
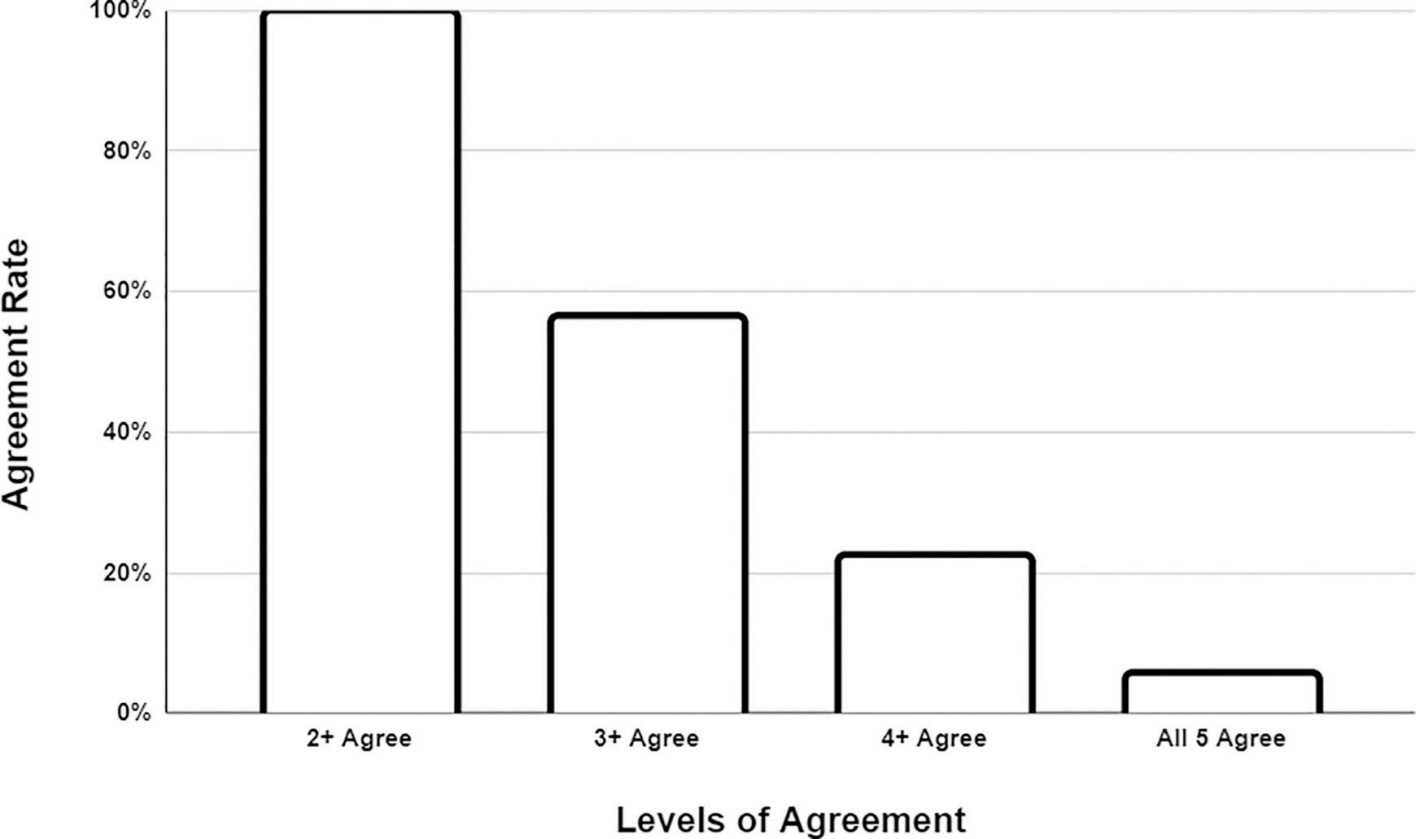
Model agreement of most likely diagnosis category. In assessing model agreement for the diagnostic category, nearly always, at least two models reached a consensus. However, it was rare for all five models to agree.

Regarding these categorized diagnoses, over 99% of the time, at least two models agreed on the initial diagnosis category. Three or more models reached a consensus on the initial diagnosis 56% of the time, while four or more models agreed in only 22% of cases. Interestingly, all five models were in agreement regarding the initial diagnosis category in only 5% of instances, even though each model was presented with identical data (Fig 6).

The recommended initial test to be ordered in the emergency department often closely paralleled the initial diagnosis but frequently did not align with clinical judgment. For instance, if the model suggested the most likely diagnosis to be gastroesophageal reflux disease, it was common for the model to recommend endoscopy as the primary test to order in the emergency department.

### Gender and racial bias

There was no discernible gender or racial bias observed in terms of the most likely diagnosis or the initial test recommended. When the most likely diagnosis was cardiovascular, an ECG was consistently recommended as the initial test, regardless of gender or race. For instance, in the case of model 3.5, an ECG was recommended as the initial test 64% of the time for men and 65% of the time for women. Similarly, for African Americans, an ECG was recommended 65.0% of the time, and for non-African Americans, an ECG was also recommended 65.0% of the time.

However, there were indications of potential bias in the assignment of weights to the variables of gender and race when calculating a risk score. On average, being male and/or African American increased the risk of acute coronary syndrome according to the five models developed based on the third dataset. These weights suggested that being male increased the risk score by 3.2% (p = 0.012 vs a null hypothesis weight of 0%). Being African American increased the risk score by 1.3% on average (p = 0.008 vs a null hypothesis weight of 0%).

## Discussion

This study has identified a significant issue with ChatGPT-4: it provides highly inconsistent risk estimates, diagnostic classifications, and test ordering recommendations when presented with identical clinical data. This level of variability is substantial enough that if integrated into clinical practice, it could lead to unpredictable patient care, particularly when contrasted with well-established scoring systems like TIMI and HEART.

In direct comparisons, the mean ChatGPT-4 score was slightly but statistically significantly higher than the TIMI score despite a robust correlation between the two. However, more critically, ChatGPT-4 yielded a different score than TIMI in nearly half of instances and exhibited substantial score fluctuations when provided with identical risk data across five distinct trials. While the mean TIMI and ChatGPT-4 scores were almost identical, the wide dispersion of ChatGPT-4 scores in contrast to TIMI raises substantial concerns regarding the model’s current reliability in predicting cardiac risk.

In this study, the mean HEART score was higher than the ChatGPT-4 score. However, ChatGPT-4 displayed significant variability in the scores it assigned, leading to a different score from HEART in nearly half of the cases. Notably, in simulated patients with low-risk ChatGPT-4 scores, HEART classified them in a higher-risk category a quarter of the time. Studies have shown that HEART not only helps identify low-risk patients suitable for an outpatient workup but also identifies high-risk patients requiring rapid intervention [21]. Thus, this disagreement in both directions is concerning. If applied clinically, one out of four patients categorized as low risk by ChatGPT-4 would be categorized as moderate or high risk by HEART. This raises concerns about the potentially serious consequences of premature discharge if relying on ChatGPT-4 risk stratification.

Both TIMI and HEART rely on test results to risk-stratify patients. To assess how ChatGPT-4 approaches simulated patients at presentation, prior to any test results, it was tasked with analyzing a large range of history and physical variables related to acute nontraumatic chest pain. Once again, ChatGPT-4 provided notably different responses when presented with identical data in multiple instances. The complexity of the 44 variables led to an even wider distribution of risk scores compared to the TIMI and HEART models. Although statistically significant, the correlation of individual ChatGPT-4 models with the average model (used as a surrogate gold standard) was only moderate. When scores were normalized to a 10-point scale, the individual models differed from the average model three-quarters of the time. This substantial disagreement reinforces the hypothesis that ChatGPT-4’s risk scoring for nontraumatic acute chest pain is inconsistent and thus unreliable. Furthermore, ChatGPT-4 struggled to determine the most likely diagnostic category, with a majority of the five models (i.e., at least 3) agreeing just barely over half of the time. Additionally, the recommendations for patient workup often were illogical, as ChatGPT-4 frequently suggested upper endoscopy as the initial test when suspecting a gastrointestinal diagnosis.

In our investigation, we sought to determine if ChatGPT-4 would incorporate racial or gender bias when assessing cases of acute nontraumatic chest pain. While both African American race and male gender were considered cardiac risk factors by ChatGPT-4, the small weights assigned to these factors suggest minimal racial or gender bias. Furthermore, ChatGPT-4 did not diagnose cardiovascular disease more or less often based on race or gender and did not recommend cardiovascular testing more or less often based on race or gender.

Although analyses of other medical conditions have identified possible racial biases [28], our analysis found the opposite. Biases in LLMs can arise from the data they were trained on. In the context of evaluating acute nontraumatic chest pain in an emergency setting, gender bias was initially identified in the early 1990s [29], which led to greater awareness and mitigation of this bias in the cardiovascular and emergency medicine literature. Nevertheless, ChatGPT-4 did give extra weight to male gender, raising the possibility of a persistent small bias in the training data affecting ChatGPT-4’s behavior.

ChatGPT-4 utilizes randomized elements within its algorithm to help it better mimic natural human language variability and creativity. Two key parameters controlling this randomness are temperature, and Top P. Temperature adjusts how predictable the model’s next token (word) is, allowing more fluctuation to mirror normal human variation in language. Top P filters out statically less probable tokens, retaining only more common words. Currently, these parameters cannot be adjusted directly through the commonly used chatbot web interface.

While a degree of randomness is important in the generation of language, randomness in analyzing medical data can have harmful repercussions. Interestingly, we found that ChatGPT-4’s randomness was also applied to its data analysis. The most significant impact was on intermediate-risk scores; however, there was substantial variability across the entire distribution relative to benchmark standards. This algorithmic randomness may present significant and serious risks to human health if used clinically due to its lack of consistency and reliability.

Limitations of this investigation include the use of simulated rather than real-world clinical data and testing a single version of ChatGPT available at one point in time. Additionally, the cases focused specifically on cardiovascular risk related to acute chest pain presentations. However, real-world clinical data would not affect the primary finding that ChatGPT-4 produces inconsistent results, making it inappropriate for clinical decision-making in the evaluation of nontraumatic chest pain. The study’s strengths are the large dataset sizes analyzed, encompassing an extensive range of variables, the comparison to established risk scores, and the multiple independent trials assessing intra-rater consistency. By comprehensively evaluating ChatGPT-4’s risk stratification capacities using robust simulated case simulations, this study provides meaningful insights into its reasoning abilities and reliability for integrating complex clinical information. These findings reveal current limitations in dependability, laying the groundwork for further refinements to realize ChatGPT-4’s promise in medical decision support.

ChatGPT-4 benefits from a vast knowledge base, having been trained on the equivalent of hundreds of millions of books. While this broad knowledge base is valuable, it also introduces contradictory and conflicting information due to the diversity of its training data. The latest iteration of ChatGPT-4, at the time of this writing, allows for the creation of specialized models trained on highly curated datasets. For example, specialized GPT models could be exclusively trained on PubMed Central articles or recognized textbooks in fields like emergency medicine, such as Rosen’s Emergency Medicine [30]. This approach could potentially reduce the impact of inconsistent, garbage in—garbage out training data [31]. In addition, combining machine learning approaches, such as neural networks with expert systems [32], may be superior to either approach alone. The ability of LLMs to scan large amounts of data makes them potentially useful in rapidly reading a patient’s entire medical record and quickly identifying important health information [33]. While the issue of randomness will still need addressing, a fruitful direction for future research would combine this ability with expert systems such as TIMI and HEART to create a more accurate and robust clinical tool. Creating customized GPTs and reducing randomness parameters could revolutionize clinical applications by providing more reliable and context-specific responses to clinical inquiries.

## Conclusions

Cardiovascular risk estimates by ChatGPT-4 on large, simulated patient datasets correlate well with the well-validated TIMI and HEART scores. However, the variability of individual scores on identical risk data makes using ChatGPT-4 for cardiac risk assessment inappropriate for clinical use. To address these issues, future investigations should explore approaches such as lowering randomness parameters and developing custom GPT models trained on curated datasets.

## References

[1] McCulloch WS, Pitts W. A logical calculus of the ideas immanent in nervous activity. Bull Math Biophys. 1943;5: 115–133. 2185863

[2] Biever C. ChatGPT broke the Turing test—the race is on for new ways to assess AI. Nature. 2023;619: 686–689. doi: 10.1038/d41586-023-02361-7 37491395

[3] Vaswani A, Shazeer N, Parmar N, Uszkoreit J, Jones L, Gomez AN, et al. Attention is all you need. In: NIPS’17: proceedings of the 31st international conference on neural information processing systems. Red Hook, NY, USA: Curran Associates Inc; 2017. pp. 6000–6010.

[4] Radford A, Narasimhan K, Salimans T, Sutskever I. Improving language understanding by generative pre-training. 2018 [cited 20 Jun 2023]. Available from: https://web.archive.org/web/20230622213848/. https://www.cs.ubc.ca/~amuham01/LING530/papers/radford2018improving.pdf.

[5] Kung TH, Cheatham M, Medenilla A, Sillos C, De Leon L, Elepaño C, et al. Performance of ChatGPT on USMLE: Potential for AI-assisted medical education using large language models. PLOS Digit Health. 2023;2: e0000198. doi: 10.1371/journal.pdig.0000198 36812645 PMC9931230

[6] Lewandowski M, Łukowicz P, Świetlik D, Barańska-Rybak W. An original study of ChatGPT-3.5 and ChatGPT-4 Dermatological Knowledge Level based on the Dermatology Specialty Certificate Examinations. Clin Exp Dermatol. 2023;llad255. doi: 10.1093/ced/llad255 37540015

[7] Heston TF, Khun C. Prompt engineering in medical education. IME. 2023;2: 198–205.

[8] Cohen F, Vallimont J, Gelfand AA. Caution regarding fabricated citations from artificial intelligence. Headache J Head Face Pain. 2023. doi: 10.1111/head.14649 37873980

[9] Sharun K, Banu SA, Pawde AM, Kumar R, Akash S, Dhama K, et al. ChatGPT and artificial hallucinations in stem cell research: assessing the accuracy of generated references—a preliminary study. Ann Med Surg (Lond). 2023;85: 5275–5278. doi: 10.1097/MS9.0000000000001228 37811040 PMC10553015

[10] Silva HECD Santos GNM, Leite AF Mesquita CRM, Figueiredo PTS Stefani CM, et al. The use of artificial intelligence tools in cancer detection compared to the traditional diagnostic imaging methods: An overview of the systematic reviews. PLoS ONE. 2023;18: e0292063. doi: 10.1371/journal.pone.0292063 37796946 PMC10553229

[11] Freeman K, Geppert J, Stinton C, Todkill D, Johnson S, Clarke A, et al. Use of artificial intelligence for image analysis in breast cancer screening programmes: systematic review of test accuracy. BMJ. 2021;374: n1872. doi: 10.1136/bmj.n1872 34470740 PMC8409323

[12] Gomes B, Pilz M, Reich C, Leuschner F, Konstandin M, Katus HA, et al. Machine learning-based risk prediction of intrahospital clinical outcomes in patients undergoing TAVI. Clin Res Cardiol. 2021;110: 343–356. doi: 10.1007/s00392-020-01691-0 32583062

[13] Corey KM, Kashyap S, Lorenzi E, Lagoo-Deenadayalan SA, Heller K, Whalen K, et al. Development and validation of machine learning models to identify high-risk surgical patients using automatically curated electronic health record data (Pythia): A retrospective, single-site study. PLoS Med. 2018;15: e1002701. doi: 10.1371/journal.pmed.1002701 30481172 PMC6258507

[14] Kwon JM, Jeon KH, Kim HM, Kim MJ, Lim S, Kim K-H, et al. Deep-learning-based risk stratification for mortality of patients with acute myocardial infarction. PLoS ONE. 2019;14: e0224502. doi: 10.1371/journal.pone.0224502 31671144 PMC6822714

[15] Myers PD, Scirica BM, Stultz CM. Machine learning improves risk stratification after acute coronary syndrome. Sci Rep. 2017;7: 12692. doi: 10.1038/s41598-017-12951-x 28978948 PMC5627253

[16] Zhang X, Wang X, Xu L, Liu J, Ren P, Wu H. The predictive value of machine learning for mortality risk in patients with acute coronary syndromes: a systematic review and meta-analysis. Eur J Med Res. 2023;28: 451. doi: 10.1186/s40001-023-01027-4 37864271 PMC10588162

[17] Aziz F, Malek S, Ibrahim KS, Raja Shariff RE, Wan Ahmad WA, Ali RM, et al. Short- and long-term mortality prediction after an acute ST-elevation myocardial infarction (STEMI) in Asians: A machine learning approach. PLoS ONE. 2021;16: e0254894. doi: 10.1371/journal.pone.0254894 34339432 PMC8328310

[18] Antman EM, Cohen M, Bernink PJ, McCabe CH, Horacek T, Papuchis G, et al. The TIMI risk score for unstable angina/non-ST elevation MI: A method for prognostication and therapeutic decision making. JAMA. 2000;284: 835–842. doi: 10.1001/jama.284.7.835 10938172

[19] Ke J, Chen Y, Wang X, Wu Z, Chen F. Indirect comparison of TIMI, HEART and GRACE for predicting major cardiovascular events in patients admitted to the emergency department with acute chest pain: a systematic review and meta-analysis. BMJ Open. 2021;11: e048356. doi: 10.1136/bmjopen-2020-048356 34408048 PMC8375746

[20] Al-Zaiti SS, Faramand Z, Alrawashdeh MO, Sereika SM, Martin-Gill C, Callaway C. Comparison of clinical risk scores for triaging high-risk chest pain patients at the emergency department. Am J Emerg Med. 2019;37: 461–467. doi: 10.1016/j.ajem.2018.06.020 29907395 PMC6286698

[21] Six AJ, Backus BE, Kelder JC. Chest pain in the emergency room: value of the HEART score. Neth Heart J. 2008;16: 191–196. doi: 10.1007/BF03086144 18665203 PMC2442661

[22] Laureano-Phillips J, Robinson RD, Aryal S, Blair S, Wilson D, Boyd K, et al. HEART Score Risk Stratification of Low-Risk Chest Pain Patients in the Emergency Department: A Systematic Review and Meta-Analysis. Ann Emerg Med. 2019;74: 187–203. doi: 10.1016/j.annemergmed.2018.12.010 30718010

[23] Six AJ, Cullen L, Backus BE, Greenslade J, Parsonage W, Aldous S, et al. The HEART score for the assessment of patients with chest pain in the emergency department: a multinational validation study. Crit Pathw Cardiol. 2013;12: 121–126. doi: 10.1097/HPC.0b013e31828b327e 23892941

[24] Poldervaart JM, Reitsma JB, Backus BE, Koffijberg H, Veldkamp RF, ten Haaf ME, et al. Effect of Using the HEART Score in Patients With Chest Pain in the Emergency Department. Ann Intern Med. 2017;166: 689–697. 28437795 10.7326/M16-1600

[25] Hess EP, Agarwal D, Chandra S, Murad MH, Erwin PJ, Hollander JE, et al. Diagnostic accuracy of the TIMI risk score in patients with chest pain in the emergency department: a meta-analysis. CMAJ. 2010;182: 1039–1044. doi: 10.1503/cmaj.092119 20530163 PMC2900327

[26] Lang TA, Altman DG. Basic statistical reporting for articles published in biomedical journals: the “Statistical Analyses and Methods in the Published Literature” or the SAMPL Guidelines. Int J Nurs Stud. 2015;52: 5–9. doi: 10.1016/j.ijnurstu.2014.09.006 25441757

[27] Diamond GA, Forrester JS. Analysis of probability as an aid in the clinical diagnosis of coronary-artery disease. N Engl J Med. 1979;300: 1350–1358. doi: 10.1056/NEJM197906143002402 440357

[28] Omiye JA, Lester JC, Spichak S, Rotemberg V, Daneshjou R. Large language models propagate race-based medicine. npj Digital Med. 2023;6: 195. doi: 10.1038/s41746-023-00939-z 37864012 PMC10589311

[29] Heston TF, Lewis LM. Gender bias in the evaluation and management of acute nontraumatic chest pain. The St. Louis emergency physicians’ association research group. Fam Pract Res J. 1992;12: 383–389. 1481708

[30] Walls R, Hockberger R, Gausche-Hill M. Rosen’s emergency medicine: concepts and clinical practice: 2-volume set. 10th ed. Philadelphia: Elsevier; 2022.

[31] MSMW. Can GIGO be eliminated? West J Med. 1979;130: 366–367. 18748413 PMC1238635

[32] Heston TF, Norman DJ, Barry JM, Bennett WM, Wilson RA. Cardiac risk stratification in renal transplantation using a form of artificial intelligence. Am J Cardiol. 1997;79: 415–417. doi: 10.1016/s0002-9149(96)00778-3 9052342

[33] Buevara M, Chen S, Thomas S, Chaunzwa TL, Franco I, Kann BH, et al. Large language models to identify social determinants of health in electronic health records. NPJ Digit Med. 2024;7:6. doi: 10.1038/s41746-023-00970-0 38200151 PMC10781957

